# 10-Year Effects of the 13-Valent Pneumococcal Conjugate Vaccine in Patients with Chronic Obstructive Pulmonary Disease and Stable Angina Pectoris

**DOI:** 10.3390/vaccines13101000

**Published:** 2025-09-25

**Authors:** Galina L. Ignatova, Sergey N. Avdeev, Vladimir N. Antonov, Elena V. Blinova, Mikhail V. Osikov

**Affiliations:** 1 Federal State Budgetary Educational Institution of Higher Education, South-Ural State Medical University, Ministry of Healthcare of the Russian Federation, 454092 Chelyabinsk, Russia; iglign@mail.ru (G.L.I.);; 2 Department of Pulmonology, I.M. Sechenov First Moscow State Medical University (Sechenov University), Ministry of Healthcare of the Russian Federation, 119048 Moscow, Russia

**Keywords:** COPD, CHD, PCV, pneumococcal conjugate vaccine, vaccine prophylaxis

## Abstract

**Background**: COPD and stable angina are common in older adults, increasing the risk of respiratory and cardiovascular complications. Pneumococcal vaccination is recommended to reduce this burden. This study evaluated the 10-year impact of 13-valent pneumococcal conjugate vaccine (PCV13) on community-acquired pneumonia (COPD), COPD exacerbations, hospitalizations, and survival in this cohort. **Methods**: A total of 483 male patients with COPD and/or stable angina received a single dose of PCV13 and were divided into three groups: Group 1 (*n* = 140): vaccinated with COPD; Group 2 (*n* = 167): vaccinated with COPD and stable angina; and Group 3 (*n* = 176): unvaccinated with COPD. Primary endpoints were CAP cases, COPD exacerbations, and hospitalizations; the secondary endpoint was survival. Analysis used generalized linear models, Cox regression, and Kaplan–Meier survival curves. **Results**: PCV13 significantly reduced CAP in patients with COPD alone but not in those with comorbid angina. Although CAP, exacerbations, and hospitalizations increased over time, vaccinated groups consistently showed lower rates than the unvaccinated group. Survival was higher in both vaccinated groups over 10 years. **Conclusions**: PCV13 was associated with a reduced risk of CAP, COPD exacerbations, hospitalizations, and improved survival in older adults with COPD and stable angina. These findings support the vaccine’s potential to improve outcomes in multimorbid populations and its inclusion in clinical guidelines and adult immunization programs for high-risk older adults.

## 1. Introduction

Chronic obstructive pulmonary disease (COPD) and stable angina pectoris are prevalent comorbidities in older adults, having overlapping risk factors, such as smoking, aging, and systemic inflammation [1,2,3]. Individuals with COPD have a 24% higher risk of developing coronary heart disease (CHD) compared to those without COPD [1]. Cardiovascular diseases (CVDs), particularly ischemic heart disease and stroke, remain the leading global cause of death and disability, with cases nearly doubling from 271 million in 1990 to 523 million in 2019, and annual deaths rising from 12.1 million to 18.6 million over the same period [4]. In the US, heart disease was the leading cause of death, while COPD ranked fifth, underscoring combined burden of both chronic diseases on public health [5].

COPD affects an estimated 392 million people globally, with prevalence rates ranging from 6.0% to 11.8% depending on diagnostic criteria, and is more common among men, older adults, and smokers [6,7,8,9,10]. Over 80% of cases occur in low- and middle-income countries, where access to preventive care may be limited [8]. The disease is associated with frequent exacerbations, hospitalizations, and progressive decline in lung function, 3.3 million deaths and 74.4 million disability-adjusted life years (DALYs) annually [11]. Severe exacerbations are linked to a 64% increase in COPD-related mortality, and many patients also exhibit coronary artery calcification and stenosis that are key risk factors for CHD [12,13].

As COPD advances, it can lead to pulmonary hypertension, which in turn contributes to right ventricular dysfunction, arrhythmias, and further cardiovascular complications [14]. Although pulmonary hypertension therapies can improve hemodynamics and reduce symptoms, they do not significantly enhance exercise capacity or oxygenation [14]. The combined impact of cardiac and pulmonary impairment results in reduced physical activity and diminished quality of life [14]. Moreover, CHD, hypertension, and declining gas exchange capacity are strong predictors of mortality in COPD, while worsening airflow limitation (FEV1 decline) is associated with increased exacerbation risk [15]. These findings emphasize the importance of comprehensive cardiovascular assessment in COPD management.

Given the shared pathophysiology and poor outcomes associated with COPD and CHD, preventive strategies are essential. Vaccination has emerged as a key component of comprehensive care. Pneumococcal conjugate vaccines (e.g., PCV13, PCV15, PCV20, and PCV21) are recommended for older adults aged ≥ 65 years or those with comorbidities, such as COPD, to prevent invasive pneumococcal diseases (IPDs), community-acquired pneumonia (CAP), COPD exacerbations, and hospitalizations [10]. Additionally, pneumococcal vaccination, influenza and COVID-19 vaccination are recommended for patients with CHD, as infections like pneumococcal infection can precipitate acute cardiac decompensation [16].

Recent findings have demonstrated the clinical benefits of PCV13 vaccination in older adults, particularly in reducing CAP and pneumonia [17,18,19]. The CAPiTA trial showed that PCV13 significantly reduced vaccine-type pneumococcal CAP and IPD, though it did not demonstrate protection against all-cause CAP [17]. Real-world observational studies by Lewnard et al. and McLaughlin et al. reported modest vaccine effectiveness (VE) against all-cause pneumonia and lower respiratory tract infections (LRTI) in general older adult populations over short follow-up periods of 3 to 4 years. These studies, while important, did not assess long-term outcomes or stratify by comorbid conditions, such as COPD and CVD. In contrast, recent findings from a 10-year prospective study, involving 362 male COPD patients, demonstrated that those vaccinated with PCV13 had significantly lower mortality, improved lung function, and fewer exacerbations and hospitalizations compared to unvaccinated controls [20]. However, the long-term impact of PCV13 in patients with overlapping chronic respiratory and cardiovascular conditions remains underexplored. Therefore, the present study aimed to evaluate the long-term effects of PCV13 on reducing CAP cases, COPD exacerbations, and hospitalizations and improving survival in older adults with COPD and stable angina pectoris over 10 years. The findings may provide further evidence that PCV13 not only mitigates respiratory and cardiovascular complications but also improves overall survival in this high-risk population.

## 2. Materials and Methods

**Figure 1:**
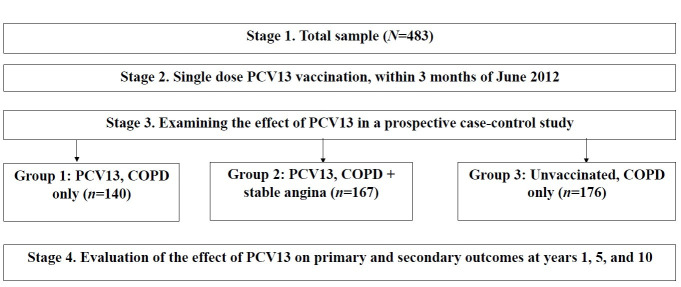
Study Design Diagram. The COPD and CHD group includes participants with COPD and stable angina pectoris. Abbreviations: COPD—Chronic Obstructive Pulmonary Disease; CHD—Coronary Heart Disease; PCV13—13-valent Pneumococcal Conjugate Vaccine.

This prospective, long-term observational study began in 2012 and remains ongoing (Figure 1). A total of 483 male outpatients with COPD and stable angina pectoris were enrolled from the patient registry of the Regional Pulmonology Center, a specialized pulmonology center, in Chelyabinsk, Russian Federation.

Eligibility Criteria: Participants were men aged ≥ 40 years with clinically confirmed COPD [21] and coronary artery disease [22]. All were current or former smokers with a minimum 10 pack-year history, capable of reproducible spirometry [23] and had a left ventricular ejection fraction > 35% on echocardiography. Inclusion required stable angina (functional classes II–IV), no prior pneumococcal vaccination, and willingness to attend follow-up visits and complete study procedures.

Exclusion Criteria: Participants were excluded if they were <40 years old or had other significant respiratory diseases (e.g., asthma, tuberculosis, bronchiectasis), decompensated pulmonary heart disease, or severe conditions affecting disease progression. Additional exclusions included Grade II arterial hypertension (i.e., systolic blood pressure > 160 mmHg), body mass index > 30 kg/m^2^, anemia (Hb < 119 g/L), systemic corticosteroid use within 3 months, malignancy within 5 years, severe psychiatric disorders, and any acute infectious or cardiovascular condition at the time of vaccination.

Study Groups: Participants were stratified into three groups: Group 1: Vaccinated with PCV13, COPD only (*n* = 140); Group 2: Vaccinated with PCV13, COPD + stable angina (*n* = 167); and Group 3: Unvaccinated, COPD only (*n* = 176). The mean age at baseline was 62.64 years (IQR: 58.12–68.76). Follow-up assessments were conducted at years 1, 5, and 10. By year 10, survival rates were 74 (Group 1), 72 (Group 2), and 34 (Group 3).

Vaccination Protocol: PCV13 was selected based on WHO 2011 recommendations and Russian Ministry of Health Order #1122 (6 December 2021) [24,25,26], which prioritize PCV13 for adults with chronic conditions. PPSV23 was not used due to its lower immunogenicity in COPD patients and the logistical challenges of monitoring revaccination over a 10-year period [20,27,28].

Gender Selection Rationale: Only male participants were included to eliminate hormonal variability, particularly the influence of female sex hormones on COPD progression [29].

Ethical Considerations: The study complied with Good Clinical Practice (GCP) standards and the Declaration of Helsinki. Ethical approval was granted by the Local Ethics Committee of the Regional Clinical Hospital #4 (Protocol #8 from 21 October 2012). All participants provided verbal and written informed consent.

Sample Size Calculation: Using Poisson regression with 95% confidence and 80% power, a 50% reduction in the dependent variable was expected in vaccinated groups [30]. The minimum sample size of 401 was increased by 20% for multivariate analysis, resulting in a final target of 481 participants. A total of 483 were enrolled.

### 2.1. Procedures

COPD and stable angina pectoris were diagnosed according to the international guidelines [21,22]. All participants were pneumococcal vaccine-naïve at enrollment, which occurred over a three-month period beginning in June 2012. Those in the vaccine group received a single dose of PCV13 and were monitored for post-vaccination adverse events. No serious adverse events were reported.

Participants underwent follow-up every three months, including: (1) clinical evaluations, (2) spirometry using MIR SPIROLAB I device (MIR, Rome, Italy), (3) oxygen saturation (SpO_2_) measured with the MD300 C2 pulse oximeter (Beijing, China), (4) electrocardiography (Schiller, Linz, Austria) via the 6-channel Sardiovitat-1 system (Schiller, Austria) after 10 min of rest, (5) echocardiography (ECHO) using the Vivid-E9 ultrasound system (General Electric, Milwaukee, WI, USA), (6) dyspnea assessment using the modified Medical Research Council (mMRC) scale (0–4 points) [31,32], (7) exercise tolerance evaluated by the 6-min walk distance (6MWD) test [33].

Comprehensive data analyses were conducted at years 1, 5, and 10. All results were recorded in the Unified Medical Information System [34]. Participants received standard therapy for COPD and stable angina pectoris in accordance with national and international guidelines [21,22]. Infectious disease monitoring, including influenza, was conducted throughout the study and documented in observation charts and the Unified Medical Information System. No severe or clinically significant infections were reported that could have affected disease progression or outcomes. Influenza vaccination was administered routinely based on epidemiological indications.

The primary endpoint was the incidence of community-acquired pneumonia (CAP), COPD exacerbations, and hospitalization rates over a 10-year follow-up. The secondary endpoint was 10-year survival.

### 2.2. Statistical Analysis

Statistical analysis was performed using R (v4.1.2) and Python (v3.11.5). Descriptive statistics were reported as medians with Q25 and Q75. Group comparisons were performed using the Kruskal–Wallis test, with multiple comparisons adjusted via the Benjamini–Hochberg procedure. Pearson’s correlation was applied to normally distributed variables. Incidence of primary outcomes per 1000 patients was computed as the number of cases in each group divided by the number of participants in each group and multiplied by 1000.

Generalized linear models with Poisson distribution assessed the effect of vaccination status on primary outcomes (CAP, exacerbations, hospitalizations), with model fit evaluated using overdispersion and likelihood ratio tests. Survival analysis was performed using Cox proportional hazard regression, with model quality assessed using the log-likelihood ratio tests and the concordance index. Kaplan–Meier survival curves were generated using the lifeline package.

## 3. Results

Baseline characteristics are presented in Table 1. The study enrolled 483 male patients, including 307 who were vaccinated with PCV13 and 176 who were unvaccinated. Vaccinated participants with COPD and stable angina had a shorter COPD history (median [Q25–Q75]: 9 [7–10.50] vs. 13 [11–15] vs. 13 [11–15], *p* = 0.012), lower oxygen saturation (88% [86–89] vs. 91 [90–92] vs. 94 [92–95], *p* = 0.021), reduced FEV1 (46 [35–58] vs. 48 [30–51] vs. 51 [47.8–56], *p* = 0.032), and a shorter 6-min walk distance (217 [190–245] vs. 230 [190–300] vs. 218 [190–240], *p* = 0.014) compared to vaccinated participants with COPD and unvaccinated participants, respectively. No significant differences were observed in age, smoking history, COPD stage, angina class, or rates of pneumonia, exacerbations, hospitalizations, or dyspnea scores.

**Figure 2:**
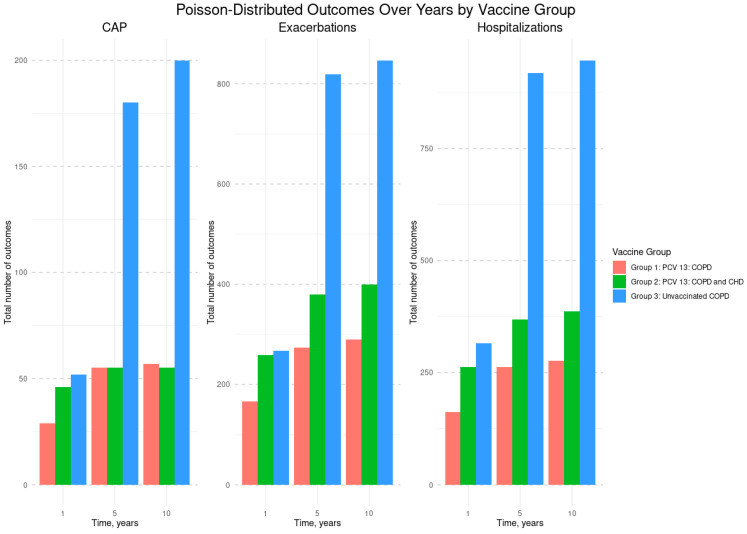
Total number of CAP, COPD exacerbations, and hospitalizations by vaccine group and by year. Abbreviations: CAP—Community-Acquired Pneumonia; COPD—Chronic Obstructive Pulmonary Disease; CHD—Coronary Heart Disease. The COPD and CHD group includes participants with COPD and stable angina pectoris; PCV13—13-valent Pneumococcal Conjugate Vaccine.

**Figure 3:**
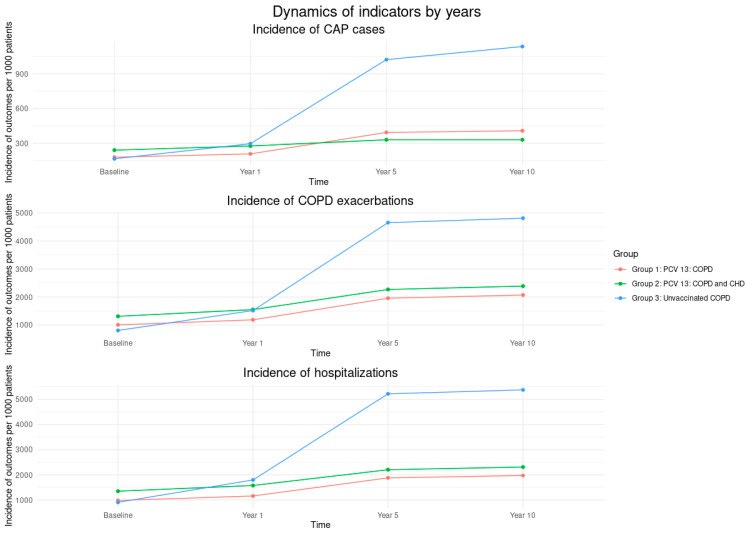
Incidence of CAP, COPD exacerbations, and hospitalizations per 1000 patients by vaccine group and by year. Abbreviations: CAP—Community-Acquired Pneumonia; COPD—Chronic Obstructive Pulmonary Disease; CHD—Coronary Heart Disease. The COPD and CHD group includes participants with COPD and stable angina pectoris; PCV13—13-valent Pneumococcal Conjugate Vaccine.

Figure 2 shows that Group 3 (unvaccinated COPD patients) had the highest total number of CAP, COPD exacerbations, and hospitalizations in years 1, 5, and 10 than Groups 1 and 2 (vaccinated with PCV13). Figure 3 presents the incidence rates of CAP, COPD exacerbations and hospitalizations per 1000 patients over time. Across all outcomes, Group 3 (unvaccinated COPD patients) consistently showed the highest incidence at each time point, while Groups 1 and 2 (vaccinated with PCV13) maintained lower and relatively stable rates throughout the follow-up period.

Table 2 reports the results of separate generalized linear models for the risk of CAP, COPD exacerbations, and hospitalizations. Vaccination with PCV13 was significantly associated with fewer CAP cases in COPD-only patients (beta coefficient [95% CI]: −0.38 [−0.72, −0.03], *p* = 0.032) but not in those with both COPD and stable angina pectoris (−0.18 [−0.45, 0.09], *p* = 0.192). However, for COPD exacerbations, vaccination reduced the risk of COPD exacerbations for vaccinated individuals with COPD (−0.37 [−0.45, −0.29], *p* < 0.0001) and those vaccinated with COPD and stable angina groups (−0.30 [−0.41, −0.20], *p* < 0.0001) compared to unvaccinated individuals. Similarly, for hospitalizations, vaccination significantly diminished the risk of hospitalizations for vaccinated with COPD (−0.69 [−0.77, −0.61], *p* < 0.0001) and vaccinated with COPD and stable angina groups (−0.76 [−0.85, −0.67], *p* < 0.0001) compared to unvaccinated groups. Over time, the risk of CAP, exacerbations, and hospitalizations increased significantly (all *p*-values < 0.0001 for years 5 and 10). Interaction effects revealed that both vaccinated groups had significantly fewer cases at years 5 and 10 across all models compared to unvaccinated participants at baseline (*p* < 0.0001). Baseline values for each outcome were significant predictors of future events.

**Figure 4:**
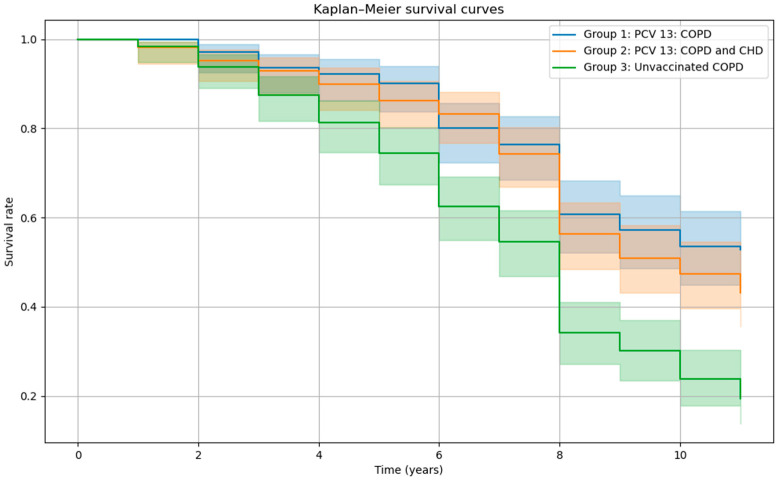
Kaplan–Meier survival curves by vaccine group and by year. Abbreviations: COPD—Chronic Obstructive Pulmonary Disease; CHD—Coronary Heart Disease. The COPD and CHD group includes participants with COPD and stable angina pectoris; PCV13—13-valent Pneumococcal Conjugate Vaccine.

Cox proportional hazards modeling showed that both vaccinated groups had significantly lower mortality risks compared to the unvaccinated group. Group 1 (vaccinated COPD patients) had an odds ratio (OR) of 0.48 (95% CI: 0.35–0.65), and Group 2 (vaccinated patients with COPD and stable angina) had an OR of 0.56 (0.43–0.73), all *p*-values < 0.05. Older age was significantly associated with higher mortality risk (OR: 1.04, 95% CI: 1.01–1.06, *p* < 0.05). Kaplan–Meier survival curves (Figure 4) demonstrated consistently higher survival probabilities over 10 years in both vaccinated groups compared to the unvaccinated group, with the unvaccinated group showing the steepest decline in survival.

The leading cause of death in Group 3 (unvaccinated, COPD) was CAP (61.2%), followed by progressive respiratory failure (31.6%). In contrast, Group 2 (vaccinated with COPD and stable angina) had more diverse causes, with stable angina pectoris (23.2%) and myocardial infarction (6.5%) contributing significantly. Group 1 (vaccinated with COPD) had the lowest mortality from CAP (5.5%) and the highest proportions of deaths attributed to other causes (81.7%).

## 4. Discussion

Our 10-year prospective observational study demonstrated that PCV13 vaccination was associated with significantly lower rates of CAP, COPD exacerbations, hospitalizations and improved survival in older male patients with and/or stable angina pectoris. At baseline, patient groups showed no statistically significant differences in key clinical and functional parameters, and all received optimized standard therapy in a specialized pulmonology center. Notably, vaccinated patients with both COPD and stable angina had slightly shorter COPD history duration, lower oxygen saturation, reduced FEV1, and a shorter 6-min walk distances compared to other groups, indicating a more severe health status due to comorbidity. These baseline characteristics, along with consistent treatment protocols, support a confident assessment of the vaccine’s impact on clinical outcomes and survival. In the unvaccinated group, survival declined progressively from year 1, with marked reductions by years 5 and 10. In contrast, vaccinated patients (COPD only or COPD and stable angina) did not demonstrate this pattern, suggesting a protective effect of PCV13 over 10 years.

Our study findings align with and extend previous research. The CAPiTA trial by Bonten et al. demonstrated that PCV13 significantly reduced vaccine-type pneumococcal CAP, non-bacterial CAP, and IPD over a 4-year period, with VE reaching 45.6% for vaccine-type CAP and 75% for IPD [17]. However, CAPiTA did not show significant protection against all-cause CAP. In contrast, our study found that PCV13 was associated with reduced rates of all-cause CAP and broader clinical outcomes, including COPD exacerbations, hospitalizations, and survival over a decade of follow-up.

Real-world studies by Hsiao et al. and McLaughlin et al. reported modest VE estimates of 8.8–10.0% against all-cause pneumonia and LRTI in older adults over 3 years [18,35]. Hsiao’s retrospective cohort study found a 10.0% VE against hospitalized pneumonia and 9.4% against LRTI [35]. Lewnard’s self-matched cohort reported 9.5% VE against medically attended LRTI and 8.8% against pneumonia [18]. These studies, while methodologically robust, did not assess long-term survival or comorbidity-specific outcomes.

Additionally, McLaughlin et al. conducted a large-scale retrospective analysis that found the VE of 72.8% against CAP hospitalizations in older adults [19]. Their findings support the broader public health value of adult pneumococcal vaccination, particularly in the context of pediatric herd immunity. In contrast to these studies [18,19,35], our research targeted a high-risk subgroup with chronic respiratory and cardiovascular conditions and used a prospective design with 10-year follow-up. This allowed for a more comprehensive evaluation of sustained vaccine impact. Importantly, PCV13 significantly reduced CAP in COPD-only patients but not in those with comorbid angina, suggesting that cardiovascular comorbidity may attenuate VE, possibly due to competing risks or altered immune response. This nuance has not been explored in prior studies.

Our study also expands the scope of clinical endpoints by including COPD exacerbations and survival, which are critical clinical outcomes in chronic disease management. The consistent benefit observed in vaccinated groups, despite increasing disease burden over time, reinforces the clinical relevance of pneumococcal vaccination. These findings support the inclusion of PCV13 in adult national immunization programs and clinical guidelines, particularly for high-risk populations, such as those with COPD and cardiovascular comorbidities. The reduced effectiveness in patients with stable angina underscores the need for tailored vaccination strategies and further research into immune modulation in comorbid patients.

This study benefits from a clearly defined cohort, high-risk cohort and a long-term follow-up period of 10 years, allowing for robust evaluation of age-related risks and vaccine impact over time. The mean age of 71 years among surviving participants underscores the clinical relevance of findings to aging populations. Unlike previous studies with shorter follow-up durations (e.g., 3–4 years) [18,19,35], our study’s extended timeline allowed for assessment of sustained clinical outcomes, including survival.

Conducting the study in a specialized pulmonology center, a tertiary care facility, ensured consistent outpatient monitoring and standardized treatment protocols. This setting enabled precise tracking of hospitalizations and exacerbations, allowing us to isolate the long-term impact of PCV13 on improved outcomes. In contrast to the CAPiTA trial [17], which focused on vaccine-type pneumococcal strains in a randomized setting, our study evaluated all-cause CAP and broader endpoints in a real-world population with COPD and stable angina. This demonstrates the importance of our findings in older adults with comorbidities. Additionally, by integrating survival analysis and stratifying by comorbidity, our study provides novel insights into how PCV13 performs in patients with overlapping chronic conditions—a research gap not addressed in prior research. These strengths position our findings as a valuable complement to existing evidence and support the inclusion of PCV13 in immunization strategies for high-risk groups.

This prospective observational study was not randomized or blinded, which may introduce potential bias. However, it was designed to provide practical insights for a broad medical audience, including general practitioners who frequently manage patients with comorbid conditions, such as COPD and stable angina pectoris. While the cohort included only male participants, it reflects a representative segment of the general population—older adults residing in a defined geographic area and receiving standardized medical care. Despite these limitations, the study remains clinically relevant given the global and national burden of COPD and coronary heart disease.

## 5. Conclusions

PCV13 was associated with a reduced risk of CAP, COPD exacerbations, hospitalization rates, and improved long-term survival in patients with COPD and stable angina pectoris. These findings support the clinical value of PCV13 and reinforce its prioritization in adult immunization programs and clinical guidelines for individuals with chronic respiratory and cardiovascular conditions.

Beyond its role in preventing infectious complications, PCV13 may contribute to mitigating disease progression and supporting long-term survival in high-risk populations. Future research is warranted to validate these findings across diverse populations and genders. Additionally, research should explore the immunological mechanisms linking pneumococcal infection to adverse cardiac events and investigate how comorbidities may modulate vaccine responsiveness in older adults. Evaluating the long-term cost-effectiveness of widespread PCV13 immunization in comorbid populations may be essential to inform sustainable public health strategies.

## Figures and Tables

**Table 1:** Study participant characteristics by vaccination status.

Variables	Vaccinated PCV13, *n* = 307		Unvaccinated, *n* = 176	*p*-Values
Group	COPD	COPD + Stable Angina	COPD	
**Sample size, *n***	140	167	176	0.123
**Age (years), median (Q25–Q75)**	61 58–64	62 59–65	65 61–68	0.327
**Smoking history (years), median (Q25–Q75)**	16.00 12.00–21.00	13.00 11.00–15.00	15.00 12.00–20.00	0.156
**COPD history (years), median (Q25–Q75)**	13 11–15	9 7–10.50	13 11–15	0.012
**COPD stages (GOLD), *n* (%)**				0.483
**II**	22 (15.7)	33 (19.8)	26 (14.7)	
**III**	106 (75.7)	116 (69.5)	138 (78.4)	
**IV**	12 (8.6)	18 (10.7)	12 (6.9)	
**Functional class of angina pectoris, *n* (%)**	-	-	-	0.176
**II**	-	31 (18.5)	-	
**III**	-	97 (58.1)	-	
**IV**	-	39 (23.4)	-	
**# CAP per year**	25	44	24	0.176
**# COPD exacerbations per year**	148	229	142	0.235
**# Hospitalizations per year**	141	235	159	0.312
**MRC dyspnea scale (points), median (Q25–Q75)**	3 2–3	3 2–3	3.20 3–4	0.076
**SpO_2_ (%), median (Q25–Q75)**	91 90–92	88 86–89	94 92–95	0.021
**FEV_1_ (%), median (Q25–Q75)**	48 30–51	46 35–58	51 47.8–56	0.032
**6MWD, (meters), median (Q25–Q75)**	230 190–300	217 190–245	218 190–240	0.014

**Abbreviations:** CAP—Community-Acquired Pneumonia; COPD—Chronic Obstructive Pulmonary Disease; Forced Expiratory Volume in 1 Second—FEV_1_; Medical Research Council Dyspnea Scale—MRC; Peripheral Capillary Oxygen Saturation—SpO_2_; 6-Minute Walk Distance test—6MWD; and PCV13—13-Valent Pneumococcal Conjugate Vaccine. **Notes:** Study Groups: Group #1: Vaccinated with PCV13, diagnosed with both COPD and stable angina; Group #2: Vaccinated with PCV13, diagnosed with COPD only; and Group #3: Unvaccinated, diagnosed with COPD. COPD stages were defined using the Global Initiative for Chronic Obstructive Lung Disease (GOLD) criteria.

**Table 2:** Results from generalized linear modeling for CAP, COPD exacerbations, and hospitalizations.

Model #1: CAP	Estimates (95% CI)	*p*-Value
**Group**		
Unvaccinated: COPD	reference	
PCV13 vaccinated: COPD	−0.38 (−0.72, −0.03)	0.032
PCV13 vaccinated: COPD and stable angina	−0.18 (−0.45, 0.09)	0.192
**Time**		
Year 1	reference	
Year 5	1.24 (0.98, 1.50)	<0.0001
Year 10	1.35 (1.09, 1.60)	<0.0001
**Interaction: Group × Time**		
Unvaccinated: COPD × Year 1	reference	
PCV13 vaccinated: COPD × Year 5	−0.60 (−0.97, −0.24)	0.001
PCV13 vaccinated: COPD × Year 10	−0.67 (−1.04, −0.30)	<0.0001
PCV13 vaccinated: COPD and stable angina × Year 5	−1.06 (−1.36, −0.77)	<0.0001
PCV13 vaccinated: COPD and stable angina × Year 10	−1.17 (−1.46, −0.88)	<0.0001
**CAP at baseline**	1.11 (0.86, 1.36)	<0.0001
**Model #2: COPD exacerbations**		
**Group**		
Unvaccinated: COPD	reference	
PCV13 vaccinated: COPD	−0.37 (−0.45, −0.29)	<0.0001
PCV13 vaccinated: COPD and stable angina	−0.30 (−0.41, −0.20)	<0.0001
**Time**		
Year 1	reference	
Year 5	1.12 (1.08, 1.16)	<0.0001
Year 10	1.16 (1.11, 1.20)	<0.0001
**Interaction: Group × Time**		
Unvaccinated: COPD × Year 1	reference	
PCV13 vaccinated: COPD × Year 5	−0.62 (−0.73, −0.51)	<0.0001
PCV13 vaccinated: COPD × Year 10	−0.60 (−0.71, −0.48)	<0.0001
PCV13 vaccinated: COPD and stable angina × Year 5	−0.74 (−0.81, −0.67)	<0.0001
PCV13 vaccinated: COPD and stable angina × Year 10	−0.72 (−0.80, −0.64)	<0.0001
**COPD exacerbations at baseline**	0.61 (0.46, 0.76)	<0.0001
**Model #3: Hospitalizations**		
**Group**		
Unvaccinated: COPD	reference	
PCV13 vaccinated: COPD	−0.69 (−0.77, −0.61)	<0.0001
PCV13 vaccinated: COPD and stable angina	−0.76 (−0.85, −0.67)	<0.0001
**Time**		
Year 1	reference	
Year 5	1.07 (1.038, 1.10)	<0.0001
Year 10	1.10 (1.065, 1.13)	<0.0001
**Interaction: Group × Time**		
Unvaccinated: COPD × Year 1	reference	
PCV13 vaccinated: COPD × Year 5	−0.58 (−0.68, −0.49)	<0.0001
PCV13 vaccinated: COPD × Year 10	−0.57 (−0.67, −0.46)	<0.0001
PCV13 vaccinated: COPD and stable angina × Year 5	−0.73 (−0.79, −0.67)	<0.0001
PCV13 vaccinated: COPD and stable angina × Year 10	−0.71 (−0.78, −0.65)	<0.0001
**Hospitalizations at baseline**	1.15 (1.03, 1.28)	<0.0001

**Abbreviations:** CAP—Community-Acquired Pneumonia; COPD—Chronic Obstructive Pulmonary Disease; and PCV13—13-Valent Pneumococcal Conjugate Vaccine. **Notes:** Study Groups: Group #1: Vaccinated with PCV13, diagnosed with both COPD and stable angina; Group #2: Vaccinated with PCV13, diagnosed with COPD only; and Group #3: Unvaccinated, diagnosed with COPD.

## Data Availability

Datasets generated during and/or analyzed during the current study are available from the corresponding author upon reasonable request.

## References

[1] Wang J.J. (2021). Risk of Coronary Heart Disease in People with Chronic Obstructive Pulmonary Disease: A Meta-Analysis. Int. J. Chron. Obs. Pulmon. Dis..

[2] Tsao C.W., Aday A.W., Almarzooq Z.I., Anderson C.A., Arora P., Avery C.L., Baker-Smith C.M., Beaton A.Z., Boehme A.K., Buxton A.E. (2023). Heart Disease and Stroke Statistics-2023 Update: A Report From the American Heart Association. Circulation.

[3] Virani S.S., Newby L.K., Arnold S.V., Bittner V., Brewer L.C., Demeter S.H., Dixon D.L., Fearon W.F., Hess B., Johnson H.M. (2023). 2023 AHA/ACC/ACCP/ASPC/NLA/PCNA Guideline for the Management of Patients With Chronic Coronary Disease: A Report of the American Heart Association/American College of Cardiology Joint Committee on Clinical Practice Guidelines. Circulation.

[4] Roth G.A., Mensah G.A., Johnson C.O., Addolorato G., Ammirati E., Baddour L.M., Barengo N.C., Beaton A.Z., Benjamin E.J., Benziger C.P. (2020). Global Burden of Cardiovascular Diseases and Risk Factors, 1990–2019: Update From the GBD 2019 Study. J. Am. Coll. Cardiol..

[5] National Heart Lung and Blood Institute (2023). Overall Mortality Data.

[6] López-Campos J.L., Tan W., Soriano J.B. (2016). Global burden of COPD. Respirology.

[7] GBD 2019 Diseases and Injuries Collaborators (2020). Global burden of 369 diseases and injuries in 204 countries and territories, 1990-2019: A systematic analysis for the Global Burden of Disease Study 2019. Lancet.

[8] Adeloye D., Song P., Zhu Y., Campbell H., Sheikh A., Rudan I. (2022). Global, regional, and national prevalence of, and risk factors for, chronic obstructive pulmonary disease (COPD) in 2019: A systematic review and modelling analysis. Lancet Respir. Med..

[9] Boers E., Barrett M., Su J.G., Benjafield A.V., Sinha S., Kaye L., Zar H.J., Vuong V., Tellez D., Gondalia R. (2023). Global Burden of Chronic Obstructive Pulmonary Disease Through 2050. JAMA Netw. Open.

[10] The Global Initiative for Chronic Obstructive Lung Disease (2025). Global Initiative for Chronic Obstructive Lung Disease, Global Strategy for Prevention, Diagnosis, and Management of COPD: 2025 Report.

[11] Safiri S., Carson-Chahhoud K., Noori M., Nejadghaderi S.A., Sullman M.J.M., Heris J.A., Ansarin K., Mansournia M.A., Collins G.S., Kolahi A.-A. (2022). Burden of chronic obstructive pulmonary disease and its attributable risk factors in 204 countries and territories, 1990–2019: Results from the Global Burden of Disease Study 2019. BMJ.

[12] Whittaker H., Rothnie K.J., Quint J.K. (2024). Cause-specific mortality in COPD subpopulations: A cohort study of 339 647 people in England. Thorax.

[13] Svendsen C.D., Kuiper K.K.J., Ostridge K., Larsen T.H., Nielsen R., Hodneland V., Nordeide E., Bakke P.S., Eagan T.M., Torén K. (2022). Factors associated with coronary heart disease in COPD patients and controls. PLoS ONE.

[14] Elkhapery A., Hammami M.B., Sulica R., Boppana H., Abdalla Z., Iyer C., Taifour H., Niu C., Deshwal H. (2023). Pulmonary Vasodilator Therapy in Severe Pulmonary Hypertension Due to Chronic Obstructive Pulmonary Disease (Severe PH-COPD): A Systematic Review and Meta-Analysis. J. Cardiovasc. Dev. Dis..

[15] Alter P., Lucke T., Watz H., Andreas S., Kahnert K., Trudzinski F.C., Speicher T., Söhler S., Bals R., Waschki B. (2022). Cardiovascular predictors of mortality and exacerbations in patients with COPD. Sci. Rep..

[16] McDonagh T.A., Metra M., Adamo M., Gardner R.S., Baumbach A., Böhm M., Burri H., Butler J., Čelutkienė J., Chioncel O. (2021). 2021 ESC Guidelines for the diagnosis and treatment of acute and chronic heart failure. Eur. Heart J..

[17] Bonten M.J.M., Huijts S.M., Bolkenbaas M., Webber C., Patterson S., Gault S., van Werkhoven C.H., Van Deursen A.M.M., Sanders E.A.M., Verheij T.J.M. (2015). Polysaccharide conjugate vaccine against pneumococcal pneumonia in adults. N. Engl. J. Med..

[18] Lewnard J.A., Bruxvoort K.J., Fischer H., Hong V.X., Grant L.R., Jódar L., Cané A., Gessner B.D., Tartof S.Y. (2022). Effectiveness of 13-Valent Pneumococcal Conjugate Vaccine Against Medically Attended Lower Respiratory Tract Infection and Pneumonia Among Older Adults. Clin. Infect. Dis..

[19] McLaughlin J.M., Jiang Q., Isturiz R.E., Sings H.L., Swerdlow D.L., Gessner B.D., Carrico R.M., Peyrani P., Wiemken T.L., Mattingly W.A. (2018). Effectiveness of 13-Valent Pneumococcal Conjugate Vaccine Against Hospitalization for Community-Acquired Pneumonia in Older US Adults: A Test-Negative Design. Clin. Infect. Dis..

[20] Ignatova G., Avdeev S.N., Antonov V.N., Blinova E.V. (2023). Ten-year analysis of the efficacy of vaccination against pneumococcal infection in patients with chronic obstructive pulmonary disease. Pulmonologiya.

[21] Vestbo J., Hurd S.S., Rodriguez-Roisin R. (2012). The 2011 revision of the global strategy for the diagnosis, management and prevention of COPD (GOLD)—Why and what?. Clin. Respir. J..

[22] Fox K., Garcia M.A.A., Ardissino D., Buszman P., Camici P.G., Crea F., Daly C., De Backer G., Hjemdahl P., Lopez-Sendon J. (2006). Guidelines on the management of stable angina pectoris: Executive summary: The Task Force on the Management of Stable Angina Pectoris of the European Society of Cardiology. Eur. Heart. J..

[23] Pellegrino R., Viegi G., Brusasco V., Crapo R.O., Burgos F., Casaburi R., Coates A., Van Der Grinten C.P.M., Gustafsson P., Hankinson J. (2005). Interpretative strategies for lung function tests. Eur. Heart. J..

[24] World Health Organization (2011). Global Status Report on Noncommunicable Diseases 2010.

[25] Ministry of Health of the Russian Federation (2025). Clinical Guidelines Rubricator. https://cr.minzdrav.gov.ru/.

[26] Avdeev S.N., Alyeva M.H., Baranov A.A., Bikmieva A.V., Briko N.I., Bulgakova V.A., Vishneva E.A., Gorelov A.V., Demko I.V., Dobrynina E.A. (2023). Federal Clinical Guidelines on Vaccination of pneumococcal infection in children and adults. Profil. Meditsina.

[27] Ignatova G.L., Avdeev S.N., Antonov V.N. (2021). Comparative effectiveness of pneumococcal vaccination with PPV23 and PCV13 in COPD patients over a 5-year follow-up cohort study. Sci. Rep..

[28] Avdeev S.N., Truschenko N.V., Gaynitdinova V.V., Soe A.K., Nuralieva G.S. (2018). Treatment of exacerbations of chronic obstructive pulmonary disease. Ter. Arkhiv.

[29] Tam A., Morrish D., Wadsworth S., Dorscheid D., Man S.P., Sin D.D. (2011). The role of female hormones on lung function in chronic lung diseases. BMC Women’s Health.

[30] Channouf N., Fredette M., MacGibbon B. (2014). Power and sample size calculations for Poisson and zero-inflated Poisson regression models. Comput. Stat. Data Anal..

[31] Stenton C. (2008). The MRC breathlessness scale. Occup. Med..

[32] Perez T., Burgel P.R., Paillasseur J.L., Caillaud D., Deslée G., Chanez P., Roche N. (2015). Modified Medical Research Council scale vs Baseline Dyspnea Index to evaluate dyspnea in chronic obstructive pulmonary disease. Int. J. Chron. Obs. Pulmon. Dis..

[33] Matos Casano H.A., Ahmed I., Anjum F. (2025). Six-Minute Walk Test. StatPearls.

[34] Group B. (2025). Unified Medical Information System. https://bars.group/directions/meditsinskaya-informatsionnaya-sistema2/.

[35] Hsiao A., Hansen J., Timbol J., Lewis N., Isturiz R., Alexander-Parrish R., McLaughlin J.M., Gessner B.D., Klein N.P. (2022). Incidence and Estimated Vaccine Effectiveness Against Hospitalizations for All-Cause Pneumonia Among Older US Adults Who Were Vaccinated and Not Vaccinated With 13-Valent Pneumococcal Conjugate Vaccine. JAMA Netw. Open.

